# Correlations of interpersonal sensitivity with negative working models of the self and other: evidence for link with attachment insecurity

**DOI:** 10.1186/1744-859X-13-5

**Published:** 2014-02-14

**Authors:** Koichi Otani, Akihito Suzuki, Yoshihiko Matsumoto, Naoshi Shibuya, Ryoichi Sadahiro, Masanori Enokido

**Affiliations:** 1 Department of Psychiatry, Yamagata University School of Medicine, 2-2-2 Iidanishi, Yamagata 990-9585, Japan

**Keywords:** Interpersonal sensitivity, Attachment insecurity, Working models, IPSM, RSQ

## Abstract

**Background:**

It has been suggested that interpersonal sensitivity, a personality trait associated with depression and anxiety disorders, is linked with attachment insecurity. To confirm this link, we studied the correlations of interpersonal sensitivity with working models of the self and other.

**Methods:**

The subjects were 301 healthy Japanese. Interpersonal sensitivity and working models of the self and other were assessed by the Interpersonal Sensitivity Measure (IPSM) and the Relationship Scales Questionnaire, respectively. The correlations of the IPSM total scores with the self-model or other-model scores were analyzed by the multiple regression analysis.

**Results:**

The IPSM total scores were correlated negatively with the self-model scores (*β* = −0.48, *p* < 0.001) and to a lesser extent with the other-model scores (*β* = −0.15, *p* < 0.01).

**Conclusions:**

The present study suggests that interpersonal sensitivity is correlated with negative working models of the self and other, providing evidence for its link with attachment insecurity.

## Background

Interpersonal sensitivity is defined as undue and excessive awareness of, and sensitivity to, the behavior and feelings of others [1]. Individuals with this trait are preoccupied with interpersonal relationships, vigilant to the behavior and mood of others, sensitive to perceived or actual criticism or rejection, and modified their behavior to comply with others' expectations [1]. Interpersonal sensitivity has been proposed as one of the vulnerability factors to depression [2-4] and also as an underlying personality trait in anxiety disorders [5,6]. The Interpersonal Sensitivity Measure (IPSM) developed by Boyce and Parker [1] and later modified by Boyce et al. [7] is a self-report scale with 28 items to assess this personality trait. The IPSM has four subscales, i.e., interpersonal awareness (seven items), separation anxiety (eight items), timidity (eight items), and fragile inner-self (five items). Interpersonal awareness refers to vigilance to the behavior and feelings of others, separation anxiety deals with anxiety about separation from significant others, timidity assesses lack of assertiveness for fear of upsetting others, and fragile inner-self identifies difficulty disclosing unlikable inner-self for fear of rejection. Respondents rate the degree to which they match each item on a 1- to 4-point scale, where 1 is ‘very unlike me’ and 4 is ‘very like me’.

According to Bowlby's attachment theory [8,9], the crucial roles of parents are to respond to a child's desire for care and to encourage a child to explore the world, developing secure attachment in the child, while lack of care and/or overprotection of parents creates insecure attachment in a child. Children internalize these experiences to form working models of attachment relationships. Two key features of these working models are, first, whether or not the attachment figure is judged to be the sort of person who in general responds to call for support and protection and, second, whether or not the self is judged to be the sort of person towards whom anyone, and the attachment figure in particular, is likely to respond in a helpful way. These working models of other people and of the self once formed in early childhood tend to persist relatively unchanged throughout adult life.

Subsequently, Bartholomew [10] postulated that the negativity of the self-model, i.e., an image of the self as unworthy of love and support, is externalized as dependency, i.e., need for others' acceptance to maintain a positive self-regard. Meanwhile, negativity of the other-model, i.e., an image of the other as unreliable and rejecting, is externalized as avoidance, i.e., avoidance of closeness to minimize eventual disappointment. Combinations of positivity or negativity of the two models yield four attachment styles, i.e., the secure style with positivity of both models, the dismissing style with positivity of the self-model and negativity of the other-model, the preoccupied style with negativity of the self-model and positivity of the other-model, and the fearful style with negativity of both models. Griffin and Bartholomew [11] developed the Relationship Scales Questionnaire (RSQ) to measure the four styles and the self- and other-models. The RSQ consists of 30 phrases including 18 phrases drawn from the prototypic descriptions of four styles [12], and respondents rate the degree to which they match each phrase on a 5-point scale ranging from ‘not at all like me’ to ‘very like me’. A number of items making up the secure, dismissing, preoccupied, and fearful subscales are 5, 5, 4, and 4, respectively. The self-model score was obtained by summing the ratings of the two styles with positive self-model (secure and dismissing) and subtracting the ratings of the two styles with negative self-model (preoccupied and fearful). The other-model score was obtained by summing the ratings of the two styles with positive other-model (secure and preoccupied) and subtracting the ratings of the two styles with negative other-model (dismissing and fearful).

Some researchers have suggested that there is a link between interpersonal sensitivity and attachment insecurity. Boyce and Parker [1] proposed that individuals who failed to achieve secure attachment in childhood are disposed to separation anxiety throughout life and they avoid the emergence of separation anxiety by being overly sensitive to any threat to their interpersonal bonds. Meanwhile, our previous studies showed that high interpersonal sensitivity was associated with parental overprotection [13] and affectionless control parenting [14], which is a combination of low care and high protection. These results also suggest a connection between interpersonal sensitivity and attachment insecurity. If interpersonal sensitivity is a personality trait or interpersonal style linked with attachment insecurity, it should be correlated with negativity of the self-model and/or other-model. Therefore, the purpose of this study was to examine the relationship between interpersonal sensitivity and the self- and other-models.

## Methods

Originally, 316 physically healthy Japanese were recruited from medical students and hospital staffs living in Yamagata Prefecture. Psychiatric screening was performed by interviews by well-trained psychiatrists and using a questionnaire on current or past psychiatric treatment and diagnosis. Six items selected from the Structured Clinical Interview for DSM-IV Axis I Disorders [15] were used for the psychiatric interview. They were A1 for major depressive episode, A16 for manic episode, B1 for delusions, B6 for hallucinations, E2 for alcohol abuse, and F68 for anxiety disorders. Of the 316 cases, 6 had psychiatric disorders and 9 had missing data. These 15 cases were excluded, and the remaining 301 cases were used for data analyses. Two hundred ten were men, and 91 were women. The mean ± SD of age was 32.5 ± 9.5 years. The study protocol was approved by the ethics committee of Yamagata University School of Medicine, and all subjects provided written informed consent to participate in the study.

Interpersonal sensitivity was measured by the Japanese version of the IPSM [16], whose reliability and validity have been confirmed. In the present sample, Cronbach's alphas for the interpersonal awareness, separation anxiety, timidity, and fragile inner-self subscales were 0.84, 0.82, 0.68, and 0.70, respectively. These values were somewhat higher than those reported by the originators of the IPSM, i.e., between 0.59 for the fragile inner-self subscale and 0.76 for the interpersonal awareness subscale [1]. The four attachment styles were assessed by the Japanese version of the RSQ [17]. In the present sample, Cronbach's alphas for the secure, dismissing, preoccupied, and fearful subscales were 0.53, 0.53, 0.64, and 0.66, respectively. These values were in the range reported by the originators of the RSQ, i.e., between 0.41 for the secure subscale and 0.70 for the dismissing subscale [11]. The self-model and other-model scores were calculated by the equations of Griffin and Bartholomew [11] mentioned before.

Statistical analyses were conducted by the Student's *t* test, forced-entry multiple regression analysis, and canonical correlation analysis using SPSS 14.0 J for Windows (SPSS Japan Inc, Tokyo, Japan). Because of the relatively small number of the female subjects, the multivariate analyses were conducted not in each sex but in the total subjects with sex as a confounding factor. In the multiple regression analysis, the dependent variable was the IPSM total score, and the independent variables were the self-model and other-model scores derived from the RSQ, age, and sex. Dummy variables were used for sex (female = 0, male = 1). In the canonical correlation analysis, the dependent variables were the IPSM subscale scores, and the independent variables were similar to those in the multiple regression analysis. A *p* value of less than 0.05 was considered significant.

## Results

Table 1 shows the IPSM and RSQ scores in the total, male, and female subjects.

**Table 1:** IPSM and RSQ scores in total, male, and female subjects

	**Total**	**Male**	**Female**
IPSM			
Total	67.3 ± 11.5	67.1 ± 11.0	67.7 ± 12.5
Interpersonal awareness	18.2 ± 3.6	18.0 ± 3.5	18.8 ± 3.8
Separation anxiety	18.6 ± 4.0	18.7 ± 3.9	18.4 ± 4.2
Timidity	20.3 ± 3.4	20.3 ± 3.4	20.3 ± 3.5
Fragile inner-self	10.2 ± 2.4	10.1 ± 2.3	10.3 ± 2.9
RSQ			
Secure	17.2 ± 2.8	17.4 ± 2.7	16.6 ± 3.1*
Dismissing	12.0 ± 3.0	12.4 ± 3.1	11.3 ± 2.8**
Preoccupied	10.5 ± 2.4	10.4 ± 2.4	10.8 ± 2.5
Fearful	9.9 ± 3.0	9.9 ± 2.9	10.0 ± 3.1
Self-model	8.7 ± 5.9	9.5 ± 5.7	7.0 ± 6.3**
Other-model	5.8 ± 6.8	5.6 ± 7.2	6.1 ± 5.9

The figures on the table indicate means ± SD. *IPSM* Interpersonal Sensitivity Measure, *RSQ* Relationship Scales Questionnaire. *p < 0.05; **p < 0.01.

In the multiple regression analysis, there were negative correlations between the IPSM total scores and the self-model or other-model scores, and the former correlation (*β* = −0.48, *p* < 0.001) was stronger than the latter correlation (*β* = −0.15, *p* < 0.01). Table 2 shows the canonical correlation analysis between the IPSM subscales and the working models. All IPSM subscales had strong negative correlations with the self-model and additionally weak negative correlations with the other-model.

**Table 2:** Canonical correlation analysis between IPSM subscales and working models

	**Canonical variates**			
	**First**	**Second**	**Third**	**Fourth**
Interpersonal awareness	0.893	−0.359	−0.071	0.261
Separation anxiety	0.862	0.206	0.460	0.061
Timidity	0.541	0.066	0.229	0.807
Fragile inner-self	0.779	0.539	−0.264	0.183
Self-model	−0.917	0.324	−0.012	−0.230
Other-model	−0.515	−0.727	0.394	−0.225
Age	−0.387	−0.168	−0.135	0.897
Sex	−0.098	0.496	0.834	0.221
Canonical correlation coefficient	0.633***	0.210**	0.181*	0.015

The figures on the table show canonical loadings. *IPSM* Interpersonal Sensitivity Measure. *p < 0.05; **p < 0.01; ***p < 0.001.

## Discussion

In the present study, the IPSM total scores were negatively correlated with the self-model and other-model scores. These results suggest a connection of interpersonal sensitivity with negative working models of the self and other. Therefore, the present study provides evidence for a link of interpersonal sensitivity with attachment insecurity. It is no wonder that the description of an individual with high interpersonal sensitivity resembles those of an insecurely attached individual such as ‘lives in constant anxiety lest he or she loses his or her attachment figure’ [8] and ‘is always prone to separation anxiety, tends to be clinging, and is anxious about exploring the world’ [9]. Also, the present results are in line with our previous results suggesting that interpersonal sensitivity has developmental origins [13,14].

The correlation of interpersonal sensitivity with the negative self-model was stronger than that with the negative other-model. Griffin and Bartholomew [18] reported that the negative self-model was correlated with low scores of the self-esteem and self-acceptance measures. Meanwhile, our previous study [19] showed that interpersonal sensitivity was correlated with low scores of the self-directedness of the Temperament and Character Inventory, which is the concept of the self as an autonomous individual such as self-confidence and self-esteem [20]. It is considered that because of these negative self-concepts, the individuals with high interpersonal sensitivity show dependency on others to maintain a positive self-regard but are disposed to have separation anxiety and, therefore, unduly and excessively aware of and sensitive to the behavior and feelings of others [1].

As discussed above, it is suggested that most of the characteristics of interpersonal sensitivity are understandable from its connection with the negative self-model. However, the weaker but still significant correlation with the negative other-model may be associated with other characteristics of interpersonal sensitivity, e.g., avoidance of relationships due to the fear of interpersonal rejection [4].

The link of interpersonal sensitivity with attachment insecurity clarified here promotes application of attachment theory to the clinical practice of patients with depression and anxiety disorders characterized by high interpersonal sensitivity. Firstly, ‘the serious illness or death either of an attachment figure or of someone cared for, or some other form of separation from them’ [8] may be involved in the onset. Secondly, ‘to provide them a secure base from which they can explore themselves and their relationships’ [21] is the first and important task for a therapist. Thirdly, ‘to help them review and modify the representational models of attachment figures and of the self’ may be necessary and useful in psychotherapy [21].

There are three possible limitations in this study. Firstly, the psychiatric screening performed might not be sufficient to exclude subjects with psychiatric disorders. Secondly, because of the relatively small number of female subjects, whether a sex difference exists in correlation patterns between interpersonal sensitivity and the two working models could not be examined. Thirdly, the subjects were Japanese medical students or hospital staffs and, therefore, our results may not be extrapolated directly to the general population or other ethnic groups.

## Conclusions

The present study suggests that interpersonal sensitivity is correlated with negativity of working models of the self and other, providing the evidence for its link with attachment insecurity.

## Competing interests

All authors declare that they have no competing interests.

## Authors’ contributions

KO conceptualized and designed the study, collected and interpreted the data, and drafted the manuscript. AS designed the study, collected and analyzed the data, and modified the manuscript. YM, NS, RS, and ME collected the data. All authors read and approved the final manuscript.
